# Methodological Analysis of the Effect of an Anti-Bullying Programme in Secondary Education through Communicative Competence: A Pre-Test–Post-Test Study with a Control-Experimental Group

**DOI:** 10.3390/ijerph17093047

**Published:** 2020-04-27

**Authors:** Fernando González-Alonso, Francisco D. Guillén-Gámez, Rosa Mᵃ de Castro-Hernández

**Affiliations:** 1 Department of Didactics and School Organization, Faculty of Education, Pontifical University of Salamanca (UPSA), 37002 Salamanca, Spain; fgonzalezal@upsa.es; 2 Department of Didactics and School Organization, Faculty of Education, University of Almería (UAL), 04120 Almería, Spain; 3 Department of Language and Literature, Faculty of Education, Pontifical University of Salamanca (UPSA), 37002 Salamanca, Spain; rmdecastrohe@upsa.es

**Keywords:** secondary education, students, coexistence, communicative competence, bullying

## Abstract

The promotion of communicative competence in students play a key role in schools for the purpose of improving social, emotional and coexistence relationships in Secondary Education students. The development of said competence can represent a great strategy to improve conflicts in the classroom, notably bullying. We used a quasi-experimental pre-test and post-test control group design with a sample of 55 students from the city of Salamanca (Spain) to analyse the level of conflict and their perceptions about bullying during the 2017–2018 academic year. The anti-bullying programme called the Improvement of Coexistence and Communicative Competence (ICCC) programme used is. The behaviour of students based on their level of coexistence with the group of classmates was measured by the INSEBULL instrument (Bullying Assessment Instrument), which added one more dimension of own elaboration. The results showed that, even though the significant levels of conflict, they decreased substantially once the ICCC programme was applied. Furthermore, we found differences between the control and experimental groups which underlined the effectiveness of the program. Regarding gender, no differences were found in the experimental group. This study shows that the development of communicative competence in students has a significant impact on their level of coexistence with other classmates, although the results suggested the need for longitudinal implementation of the programme in order to improve school coexistence and social skills of students from the early stages of education.

## 1. Introduction

In the education context, bullying and harassment represent important global problems with negative consequences for physical and mental health [1], and teachers’ involvement is key to the comprehensive training of students [2]. Thus, it is not surprising that bullying has been the subject of numerous studies in the school context regarding public health [3], self-harm [4], sexual orientation and gender identity [5], and cyberbullying [6].

Globally, UNICEF [7] reported on the situation of violence against children between 13 and 15 years old, stating that out of a total of 130 million schoolchildren, approximately one in three have experienced bullying. In the Spanish context, the Non-Governmental Organization Save the Children [8] stated that six out of 10 Spanish children acknowledge that another classmate has insulted them in recent months, of which 22.6% affirmed it ocurred on a repeated basis and in more than a third it was carried out via mobile or the Internet. Almost 30% of children claim to have received physical blows.

Olweus et al. [9] affirm that bullying prevalence is a form of behaviour which empower aggressors over their victims. In addition, this type of conflict usually repeats itself over time. Bullying prevalence and victimisation are situations intentionally and systematically produced to another individual through improper behaviour, and involves an unequal and abnormal relationship between classmates [10]. There are different individual actors involved in bullying situation considering a school environment: a victim, an aggressor, and spectators [11]. 

The main profile of the victim or harassed individual is of a minor, verbally victimised in the schoolyard [12]. Cook et al. [13] affirm the profile of the aggressor or harasser influences in person, in acts of physical abuse, such as beatings, robberies and humiliations, against minors who have a clear weakness due to race, sexuality or gender [14]. For example, Elipe et al. [15] analysed homophobic bullying and cyberbullying with the participation of 533 Spanish high school students where they found that students identified as non-heterosexual experienced a higher level of being subjected to bullying and cyberbullying. Regarding gender, there are different studies that show that boys’ behaviour against bullying is based more on physical aggression and bullying than that of girls [16,17]. Regarding the female gender, it is they who often manifest behaviours related to insult and social isolation [18]. For this reason, schools must work with respect to the awareness of the norms and perceptions of the students, as well as on how they express themselves, since they can lay the foundation for bullying prevalence [19].

In relation to the victim’s profile, the most typical victims show a sensitive, shy, and insecure character, with low self-esteem and a negative view of themselves and their situation [20,21]. In this context, Romero-Reignier and Prado-Gascó [22] analysed the impact that any of the three roles involved in a bullying situation (victim, aggressor or observer) may have on the self-esteem of a sample composed by 290 Spanish adolescents. The results suggested that only the bullying victim dimension seems to have a negative relationship in relation to their self-esteem.

Therefore, it is reasonable to assume that, after prolonged intimidation by their classmates, victims tend to show more insecurity and lack of development of the communication competence required to communicate with their classmates in social situations [23].

### 1.1. Importance of Promoting Communication Competence to Improve School Coexistence

We consider that dialogue and communication is promoting school coexistence. Some students may lack the language skills required to respond to verbal forms of bullying [24,25]. For the development of school coexistence, communicative competence turns out to be a very effective element to favour the evolution of moral reasoning, at the same time that it fosters the adoption of perspectives, stimulating the empathic capacity and tolerance needed in all coexistence situations. The communicative competence is the set of knowledge and skills (linguistic, discursive, sociocultural and strategic) that the student must bring into play to understand and produce valid and appropriate discourses facing the situation and context of communication, as well as to the degree of formality required in each moment. Richmond and McCroskey [26] stated that perceived communicative competence is positively related to high self-esteem and social skills. For this reason, effective communication plays a protective role against victimisation, since as friendships evolve and interactions between students intensify, the development of self-esteem, empathy, and tolerance increases proportionally [23,27,28]. 

To achieve this purpose, it is necessary to have a positive perception towards understanding the arguments, feelings and points of view of other classmates through discussions and/or debates, where oral or written language is the ideal mean of thinking which, in this way, enables the feeling of belonging to the social group in the classroom. Specifically, communicative competence is related to the acquisition of language skills through the teaching–learning process with resources that promote speaking, listening, reading, and writing [29]. For this purpose, the Improvement of Coexistence and Communicative Competence (ICCC) programme was created by Castro and Alonso [30]. This project aims to improve school coexistence by becoming aware of the importance of verbal and non-verbal communication in relationships with other classmates. Through a positive use of language, they seek to prevent and resolve school conflicts. In this sense, it will be possible to form a democratic society where everyone belongs as a citizen.

### 1.2. Gender and Bullying Prevalence: How They Relate

Gender roles and sexuality are one of the factors that most influence the motives and profiles of bullying prevalence. Regarding gender roles, Chocarro and Garaigordobil [31], with a sample of 979 Spanish adolescents aged between 13 and 18 years old, found that boys showed a more aggressive profile than girls, as well as there was a higher percentage of women in the role of victims. In the same context, Aviles [32] investigated the usual profile that students occupied in situations of bullying. With a sample of 1433 Spanish students aged between 10 and 18 years old, the results showed the absence of significant differences regarding gender between profiles, since it does occur between common aggressors in favour of boys; and among habitual witnesses in favours of girls. In a broader context, Ruiz et al. [33] analysed the perception of 41 students from Girona (Spain) aged between 10 and 12 years old with respect to the different types of bullying prevalence (physical, verbal and social exclusion) based on gender. The results showed that physical bullying does occurs more in the male gender, while verbal bullying and social exclusion prevail in the female gender. Similar results were found by Ordóñez and Prado-Cabrera [34], showing that the role of victimised aggressor in both bullying and cyberbullying cases presents a higher level of involvement at the male gender level.

In the sexual context, de Stéfano-Barbero [35] reflected on the role that gender plays in homophobia. With a sample of 3236 students from different Spanish territories, he found that the female gender has a more positive perception towards bullying in contrast with male gender, as the latter believe it represents a very serious matter. Furthermore, the male gender doubled the cases of responses to the female gender, who considered bullying as something “inevitable as there are people who deserve it.”

However, Navarro et al. [36] affirm that the problem lies not in gender, but rather in gender identity, as well as gender typification measures are related to victims and aggressors. Carrera-Fernández et al. [37] study the relationship of bullying and the perception of masculinity and femininity: With a sample of 93 Spanish adolescents, the results showed how the men and women who are victims of aggression are those who in some way transgress gender limits. Similar results were found by Morales et al. [38]: With a sample of 2560 Spanish students, the results showed that the aggressors, both boys and girls, had masculine identity traits and behaviours.

Regarding this problem, it is essential that programmes and mechanisms are carried out that improve school coexistence at school, since it is in one of the settings where most cases of bullying does occur.

### 1.3. Studies Related to Anti-bullying Programmes and Programmes to Promote Communicative Competence

In the education community, training and prevention actions must be implemented to involve families and all community members [39]. A number of authors have carried out multiple studies based on the effect of intervention programmes to reduce bullying prevalence with students [40,41], with students and teachers [42,43], and with family intervention [39,44,45]. Nevertheless, very few studies have focused on improving the cohesion levels of students through the promotion of language proficiency. 

Ferrer-Cascales et al. [46] evaluated the effectiveness of the TEI programme (Tutoría Entre Iguales) which was focused on classmates tutoring in order to improve the school climate. With a control and experimental group of 2389 Spanish students, the results obtained showed a significant reduction in bullying behaviour, classmates victimisation, fighting, cyberbullying and cyber-victimisation in the experimental group after the implementation of the intervention. However, the authors did not control the effects regarding gender. With a similar design, Muñoz-Fernández et al. [47] evaluated the effectiveness of a school prevention programme called “Dat-e Adolescence” for six months. To this effect, they used a sample of 1423 students from Andalusia (Spain) aged between 11 and 19 years old. The programme was effective in reducing severe sexual dating violence and bullying victimisation. However, this study did not control gender differences. Similar results were found by McCoy et al. [48] and Siregar et al. [49].

Ortega-Barón et al. [50] evaluated the effectiveness of a bullying and cyberbullying programme called Prev@cib in a random sample of 660 adolescents aged between 12 and 17 years old living in the city of Valencia (Spain). The results presented a significant decrease in bullying and victimisation, as well as in cyberbullying in the experimental group as compared to the control group. However, they did not take into account the gender variable. In the same context, Martín and Bolaños [51] sought to know if the intervention of an anti-bullying programme improved the social competence of 206 students from Cordoba (Spain) aged between 12 and 16 years old. They used a design with control-experimental groups, where the results showed that the experimental group developed less involvement concerning the victim and aggression profile. The authors also did not take into account the gender variable. With a more consolidated design, Negi and Magre [52] evaluated the effectiveness of the CyberBullying Sensitisation Programme (CBSP) when cyberbullying awareness experienced a growth in 186 secondary school students. For this purpose, a pre-test and post-test design was used, with a control and experimental group. Statistical analysis indicated that there were significant differences between the pre-test and post-test scores obtained in the experimental group. Additionally, no significant difference was found between the experimental group and the control group prior to the programme, suggesting the effectiveness of the programme aimed at helping students to reduce cyberbullying behaviour. Similar results were found by Palladino et al. [53] and Lang [54].

However, there are hardly any intervention programmes that develop communicative competence as an anti-bullying criterion [55,56]. With pre-test and post-test designs, as well as with a control and experimental group, Epelde-Larrañaga [57] studied the effectiveness of the anti-bullying programme to reduce aggressive behaviour of 200 students from Melilla (Spain) aged between 11 and 14 years old. The programme was carried out for four months using music and reinforced with a talk to work on the communicative competence of the students where they spoke openly about their experience with bullying, cyberbullying or both of them. The results showed a decrease regarding victims, aggressors and witnesses of cyberbullying. However, they did not account for gender differences. In a similar context, Donnery [58] analysed the benefits of using dramaturgy in developing oral communication skills in order to create social awareness of the harasser and victim’s role. The dramaturgy process lasted between 8–16 weeks, and it was implemented with a sample of first-year students from Kwansei Gakuin University (Japan). The results presented that the students showed genuine interest when discussing the nature of bullying, showing a clear concern for the different agents involved (bully, victim, and spectator). In the same context, Blood and Blood [28] conducted a programme intended to address bullying prevalence. With a sample of 106 adolescents from the state of Pennsylvania (USA), divided into those who stuttered and those who did not stutter, the results showed that the adolescents who stuttered seemed to have developed successful strategies to deal with their lack of communicative confidence, highlighting the relationship between communicative competence and the risk of being bullied. Similar results were found by Lehman [59] and Chang et al. [60], evidencing that the language interaction between student–teacher or student–student in the classroom helped to decrease the probability that children will be victims of aggression.

Taking into account the few studies related to communicative competence as a bullying prevention tool, and contributing to the development of this competence to lower levels of conflict in the classroom (as well as the study regarding gender), the purpose of this study rests in the application of ICCC programme in secondary education students in order to improve coexistence in the classroom and, thus, promote interrelation, decrease conflict, and prevent bullying, in addition to promote coexistence and communication. The specific objectives of this study are: (1) to measure the level of conflict of the students and their perceptions before and after applying the ICCC programme, using the bullying dimension of the INSEBULL instrument (Bullying Assessment Instrument), as well as the creation of a new dimension on communicative competence; (2) to measure the level of conflict of the students and their perceptions in an experimental group versus a control group; and (3) to know if gender variable influences the level of conflict and perceptions before and after applying the ICCC programme. Therefore, the following hypotheses were formulated:

(1) There will not be differences regarding the scores of students during pre-test stage between both groups, as well as according to gender.

Between the pre-test and post-test of the experimental group:

(2) There will not be differences in the level of conflict of the students between the pre-test and post-test scores in the experimental group after applying the ICCC programme.
(3) There will not be differences about communication skills of students between the pre-test and post-test scores in the experimental group after applying the ICCC programme.

Between the control and experimental group once the programme has been applied:

(4) There will not be differences regarding the level of conflict of the students between the control and experimental groups’ scores after applying the ICCC programme.
(5) There will not be differences about communication skills of students between the control and experimental groups’ scores after applying the ICCC programme.

Between gender in the experimental group:

(6) There will not be differences regarding the level of conflict of the student with respect to gender in the pre-test and post-test design in the experimental group.
(7) There will not be differences about communication skills of students regarding gender in the pre-test and post-test design in the experimental group.

## 2. Method

### 2.1. Design

A quasi-experimental (there is no randomization of subjects for the control and experimental groups) and non-equivalent mixed study was conducted, adding a control group to the pre-test and post-test design, in order to control the possible internal threats of maturation and the application of the instrument. Descriptive and inferential analyses were carried out to identify differences between target groups. In the experimental group, the ICCC programme was applied. In the control group, the programme was not applied; the perceptions of the students were simply measured before and at the end of the investigation. The data was collected via a questionnaire and occurred at two time points: before starting the treatment (pre-test), and at the end of the treatment of the experimental group (post-test). Table 1 shows the list of variables used in the research.

### 2.2. Participants

Participants were selected through a non-probability intentional sampling process. The sample included 55 second-year students in secondary education from the city of Salamanca (Spain) in the academic year 2017–2018. Regarding sociodemographic characteristics, 49% of the participants were female, with a mean age of 13.26 ± 0.53; while 51% were male, with a mean age of 13.29 ± 0.60. From a methodological point of view, the control group was made up of 26 students, where 54% were female and 46% male; while the experimental group was made up of 29 students, where 48% were female and 52% male.

### 2.3. Research Ethics

The study complied with the ethical values required in the research regarding students privacy (informed consent and right to information, data protection and confidentiality), having received a favourable evaluation from both the head of the school and the teachers cloister. Even though the study was classified as minimal risk, which does not represent any type of damage to the integrity of the participants, written informed consent was obtained from their families in order to carry out the ICCC programme. Once the it was applied and the data collected, a meeting was held with family members and students in order to argue and justify the need to enhance oral skills to prevent bullying behaviors.

### 2.4. Instrument Description 

In the present study, the INSEBULL instrument was employed [61], with the aim of measuring the level of conflict and being able to prevent possible conflicts that may arise in the classroom. This was used before and after applying the ICCC programme. The psychometric properties of the instrument were acceptable. Regarding reliability, this was measured using Cronbach’s alpha with a very satisfactory level (α = 0.84). According to validity, the authors carried out content validity through the judgment of 10 experts who considered the definition of each of the dimensions using the selected items to be adequate. In addition, they carried out an Exploratory Factor Analysis (EFA) to check the internal consistency of the instrument. Eight factors were found which explained 57.10% of the true variance of the instrument. For this studio, the dimension of bullying prevalence was selected from the original instrument created by the authors, which measures the perception and awareness the student expresses as the aggressor protagonist in situations of conflict or intimidation of classmates (the original instrument consisted of six items (for example: “How often have you nicknamed your classmates?”). The reliability of this dimension was very satisfactory (α = 0.90).

In addition, a new dimension of this author’s own elaboration, called “conflict resolution” and consisting of three items, was added in order to collect the perceptions of the students regarding the tasks of the ICCC programme, which relate to four linguistic skills: listening, speaking, reading, and writing (for example: “Talk and listen to your classmates about bullying or teasing at school; do you think this would help to reduce and solve them?”). The reliability of this dimension was α = 0.69.

Consequently, the instrument used for the present investigation was made up of two dimensions, where the maximum score to be reached was 44 points. A scale was developed in order to establish criteria to identify levels of bullying prevalence. Table 2 shows the test scores according to three possible scales or levels of conflict (low aggression, medium aggression, and high aggression).

### 2.5. Procedure and Programme Description

The ICCC programme is a proposed intervention aiming to improve students’ use of language (communicative competence) to build a peaceful coexistence. To this end, the programme focuses on enhancing perceptions, skills, and values through four language skills (listening, speaking, reading, and writing). It has the following characteristics:

- It is a programme designed with a teaching structure that takes into account different basic competences of the Spanish curriculum defined by the LOMCE (Spanish Organic Law 8/2013, of 9th December, for the improvement of educational quality). The skills to be developed are the following ones: competence in linguistic communication; social and civic competences; and competencies in learning to learn.
- The programme will be based on different basic aspects: Teaching values (tolerance, equality, respect, and empathy) and attention given the diversity of the students.
- Three coherently articulated blocks are developed in the program: (1) school coexistence, (2) development of coexistence, (3) coexistence and communication. The tasks performed in each block are described below.
- It follows a constructivist model, because it fosters meaningful and autonomous learning for students.

The person responsible for implementing the programme was the teacher-tutor of Spanish Language and Literature subject and that one of Education for Citizenship course, both for the 2017–2018 academic year. Even though this teacher differs from the researchers indicated in this study, the results of the programme were not influenced by this factor. The work of the researchers during this programme was focused on helping to create the tasks and guiding the classroom teacher in the development of the sessions. The timing of the programme implementation was from October to June, where between two and three sessions were held each month, depending on the type of activity. Finally, given this factor, a total of 20 sessions was applied. The INSEBULL questionnaire was filled out by the students individually. Specify that this was completed in the first and last session. As it was a programme for 9 months, the effect of doing a pre-test/post-test was mitigated, since the time elapsed between the application of both tests was more than enough. 

The methodology used also depended on the type of task, but it was focused on individual activities, those ones completed in pairs, in collaborative groups of 4–5 students, and in large groups. Some of the activities carried out are described below and classified in three content blocks:

School coexistence:

- Task 1: creation of norms and values by groups, where each will be responsible for defending those that are not upheld in the classroom, encouraging positive perceptions in the students who do not comply with them.
- Task 2: analysis of and reflection on journalistic news concerning the school environment. Through discussion groups, students will note the highlights of the news and share possible solutions to problems orally.
- Task 3: in pairs, students will write down the qualities of their partner on a piece of paper, and then comment orally in a large group. In this way, all students will be able to see they share similar interests, thus increasing the tolerance for the diversity of opinions.

Creation of coexistence:

- Task 1: with short videos of television programmes, the students must use their ability to listen and manifest in groups the mistakes they could identify, in order to collect on a poster board the most significant elements that they must respect in a dialogue with another person.
- Task 2: the teacher will present a conflict situation, either one that has taken place in class or a hypothetical one. In small groups, the students will perform a dramatisation of the conflict; the teacher will assign a role to each member of the group, which they will have to interpret. It is recommended that the situation and characters be provided in written form. Students are allowed a few minutes to learn about the situation and the characters. At the end of the dramatisation, an oral debate is held to reflect on how they felt in the role given. Ideally, each role rotates between each member of each group.

Coexistence and communication

- Task 1: through the different conflicts that arise in the classroom, in small groups the students must reason orally and in writing about what is right. In a large group, the best solution is discussed and agreed by consensus.
- Task 2: anonymously, each student writes down a conflict that has occurred in or out of the classroom. In a large group, they will reflect on it in order to enhance the empathy of the students and enable them to put themselves in the place of others. To do this, the teacher will ask the following questions: How did they feel when their opinion did not coincide with the rest of the classmates? Has there been respect between the different opinions? Have they empathised with their classmates? Has someone changed their point of view after listening to other people?

### 2.6. Data Analysis

The data analysis included several procedures, which are detailed below:

- First, an exploratory descriptive analysis was carried out for each of the dimensions of the instrument in the experimental group, taking into account the time before and time after applying the ICCC programme.
- Second, to rule out possible differences between the groups prior to the start of the ICCCC program, pretest scores were analyzed using an ANOVA test with one group factor (experimental and control) and another gender factor (male or female). For this, the IBM SPSS V.22 (Armonk, NY, USA) software was used. Although there was no normality in the data through the Shapiro-Wilks test to perform this test, we proceeded with this since, in most of the subcategories of the variables studied, the Komogorov-Smirnov test was not violated. Furthermore, the assumption of homocedasticity of the variances was fulfilled.
- Third, to verify the effectiveness of the ICCC programme, different statistical tests were used to compare the pre-test group versus the post-test group; and the control group versus the experimental group. In addition, the non- of the assumptions that allow this type of analysis was studied. Specifically, in the pre-test and post-test the Wilcoxon test was used when comparing the scores of related samples. Between the control and experimental groups, the Mann-Whitney test was used when comparing independent samples.
- Finally, a differential analysis regarding gender is carried out, with the purpose of knowing if there are differences in the scores of the students in the experimental group, both in the pre-test and in the post-test. Regarding gender, different tests were used depending on whether there was normality in the data.

## 3. Results

The results section is divided into five sections: the first section, the perceptions of the students regarding each dimension of the instrument are descriptively analysed; in the second section, the equality between the groups before starting the ICCC programme is studied; the third section analyses the efficacy of the programme by comparing the pre-test and post-test scores of the experimental group; in the fourth section, the effectiveness of the programme between the control and experimental groups is analysed; and in the last section, gender is compared in the pre-test and post-test design.

### 3.1. Descriptive Analysis in the Experimental Group (before and after Applying the Programme)

Table 3 shows the perceptions of students in the experimental group regarding the bullying prevalence dimension, before and after conducting the ICCC programme. The mean and its corresponding standard deviation are detailed in each column.

In general, Table 4 shows a decrease in the scores of the different items, taking into account the times before and after implementing the ICCC programme. Specifically, some of the items that stood out were: “Would you be able to intimidate a classmate?” where the students’ perceptions slightly decreased after the treatment was applied (M_before_ = 1.73; M_after_ = 1.69). Regarding the item “How often have you given or called someone by their nickname?” it was observed that this was the item where students had a higher perception (M_before_ = 3.23), although there was a decrease once the programme had been applied (M_after_ = 2.90). In regard to the item “What do you usually do when one partner intimidates another?” the perceptions of the students before the programme were medium-high (M_before_ = 2.46), and, although there was a decrease after applying the programme (M_after_ = 2.28), the difference was small.

Table 4 shows the students’ perceptions of the tasks of the ICCC programme itself. It should be borne in mind that the maximum value (5 points) indicates that the tasks of the programme do “nothing” to improve conflict, while the minimum value (1 point) means that the tasks of the programme contribute “a lot” to decreasing conflict between students. In general, it was observed that all the items showed a decrease in the scores, meaning that the students determined that the programme tasks helped indeed.

Specifically, the item “take readings and analyse them” was the one that decreased the most once the programme had been applied (M_before_ = 4.00; M_after_ = 2.46). The item “talk and listen to your classmates about bullying at school” decreased by one point (M_before_ = 3.96; M_after_ = 2.96). Finally, the item “writing and analysing texts about bullying” was the item that decreased the least (M_before_ = 3.71; M_after_ = 2.57). A decrease was observed, although the scores remained medium-high.

### 3.2. Analysis of Group Matching

The normality assumption was verified in a bivariate way. Although the Shapiro-Wilk test violated the assumption, the Kolmogorov-Smirnov test did not violate it in most of the subcategories of the two variables studied. Even so, we have continued with the procedure since Levene’s equality test determined that the variance of the scores was the same between the groups: *L* (3, 51) = 0.105; *p* = 0.957, supporting the assumption of homoskedasticity. The analysis of possible pre-treatment differences (pretest) between the groups did not show any significant results, *F* (3, 51) = 1063, *p* = 0.373. Individually, the group variable was not significant, *F* (1, 51) = 0.086; *p* = 0.771; the gender variable was not significant either, *F* (1, 51) = 1.202; *p*. = 0.278; and finally, the intersection of both variables was also not significant, *F* (1, 51) = 1.943; *p* = 0.169. Therefore, the possibility that differences due to treatment could be due to previous differences between the groups was ruled out. The results indicated that there were no differences between groups or gender before starting the ICCC program.

### 3.3. Analysis between the Pre-Test and Post-Test of the Experimental Group

In order to analyse whether the ICCC programme had a significant impact, a comparison between pre-test and post-test scores in the experimental group was carried out in order to determine whether there was a decrease regarding the level of conflict. Table 5 shows a decrease in students’ perceptions regarding the level of perceived conflict in each of the dimensions, mainly in dimension number two. Wilcoxon’s test determined that there were statistically significant differences between the pre-test and the post-test scores in the bullying dimension (z = −2712; *p* < 0.05), the communication skills dimension (z = −0.365; *p* < 0.05), and the instrument total (z = −4565; *p* < 0.05). Furthermore, the calculated coefficient of determination produced a large effect size (r^2^ = 0.896).

### 3.4. Analy sis between the Control and Experimental Group once the Programme Has Been Applied

Table 6 shows the results of the comparison between the control and experimental group after applying the ICCC programme, with the aim of determining whether the experimental group showed a decrease in the level of conflict compared to the control group, where the treatment was not applied. Table 6 shows how in each of the dimensions of the instrument, as well as in the total scores, the scores of the experimental group revealed a decrease. The Mann–Whitney test determined that there were statistically significant differences in the bullying prevalence dimension (Z = 174,000; *p* < 0.05), in the communication skills dimension (Z = 89,500: *p* < 0.05), and in the instrument total (Z = 156,000; *p* < 0.05). Furthermore, eta squared (η^2^) determined a large effect size η^2^ = 0.896.

### 3.5. Analysis Regarding the Influence of Gender

Table 7 shows whether there were differences in the gender variable at the start of the study (pre-test), as well as at the end of the ICCC programme intervention (post-test), in each of the instrument’s dimensions. Although in some dimensions data showed normal outcomes, the same criteria are used for all analyses: in this case, the Mann-Whitney test. It is observed that neither the pre-test nor the post-test revealed significant differences in the level of conflict (dimension 1) and perceptions (dimension 2) of the students regarding gender.

## 4. Discussion 

The purpose of this study was to evaluate the effectiveness of the ICCC programme in improving coexistence in the secondary education classroom and, consequently, in reducing conflict and preventing bullying prevalence. The different hypotheses of the study will be discussed in the following lines.

First, the statistical results showed that there were no significant effects neither in the design groups nor in the genre before starting the ICCC programme: both student groups showed the same scores before starting the programme, confirming hypothesis 1. This result indicates that, where significant differences are found between the pre-test and post-test, these will be due to the intervention programme and not to the distribution of the student groups, since the sample was not probabilistic.

Surprisingly, the results showed the presence of differences between the pre-test and post-test in the experimental group, in both dimensions of the instrument, meaning hypothesis 2 and 3 were rejected. These results highlight the significant effect of the ICCC programme on students, which corresponds with the results found in similar studies such as those by Ferrer-Cascales et al. [46], Muñoz-Fernández [47], McCoy et al. [48], and Siregar et al. [49], among others. The development of activities through the use of communicative competence and the associated skills (writing, reading, speaking, and listening) was able to decrease their perceptions of bullying prevalence. These results corroborate those obtained by Chang et al. [60] and Lehman [59], who found that the use of language between student–teacher and student–student can help reduce the likelihood of aggression among classmates. However, it would be necessary to consider those items in which the level of conflict was minimally reduced, for example “Would you be able to intimidate a partner? This thought makes us think that bullying prevalence may reoccur, so we contemplate that this programme must continue to be applied in the following courses in order to control this kind of situation.

Through the ICCC programme, communicative activities related to the development of students’ self-esteem and empathy towards other classmates were put into practice. The results of the pre-test and post-test revealed a decrease in the levels of conflict and displayed a better climate in classroom. As found by Epelde-Larrañaga et al. [57] and Blood and Blood [28], the use of effective linguistic communication plays a protective role against bullying prevalence. Furthermore, the correct development of this competence over time not only produces a decrease in classroom conflicts, but also causes relationships between students to improve.

The positive effects of ICCC programme were consistent with our expectations, and the results found between the experimental group and the control group once the programme had been applied were significant, meaning hypothesis 4 ad 5 were rejected. Differences were found among those students who worked through problems using verbal and nonverbal communication, such as those problems related to bullying prevalence in comparison to the control group. The results corroborate the findings of studies by Palladino et al. [53], Lang [54], and Ortega-Barón et al. [50], who also found differences between control and experimental groups when applying programmes against bullying prevalence; although they contradicted the results of a study by Negi and Magre [52], which found no differences between control and experimental groups, but did find differences between the pre-test and post-test in the experimental group.

Lastly, no significant differences in gender were found when carrying out the ICCC programme. The students’ level of conflict and their perceptions were verified before and after applying the programme, but no differences were showed, affirming hypothesis 6 and 7. These results are contradictory to those obtained by Chocarro and Garaigordobil [31], Ruiz et al. [33] and Ordóñez and Prado-Cabrera [34]. Perhaps the reason for such results was due to the justification provided by Navarro et al. (2016) when they affirm that the problem does not lie in gender, but in gender identity.

## 5. Limitations

One barrier to the anti-bullying programme was focused on comply with school policy, all students were mandatory to fill out their questionnaires anonymously; only a code was used to relate the answers between the pre-test and post-test. Nevertheless, this was not enough, since the ideal is to be able to improve the levels of conflict among the students who require it. Another limitation found in this project lied on the type of sampling used. Another limitation was found on the kind of sampling employed, which lies on a convenience procedure. The last barrier identified was the absence of students on the days that the ICCC programme was held. Even so, the large number of sessions during a school year (20 sessions) makes students continue enhancing their communicative competence even if they do not attend any session.

## 6. Conclusions and Further Works

Considering the harmful effects that bullying prevalence may arise on students, the development of communicative competence in this community could have important implications for school coexistence. The results found in this study provide scientific evidence of the effectiveness of the ICCC programme. Those students who promote speech, express themselves, write and listen with tolerance of and empathy to their classmates, will develop socio-affective skills which will allow them to be more competent people in a democratic and social environment.

The study results demonstrate the effectiveness of the programme since the level of conflict among students was reduced, both in the pre-test and post-test stages, in the experimental group, and between the control and experimental groups. This data provides strong empirical support for a research-based bullying prevalence prevention programme. In addition, they provide guidance to educators and families on possible educational strategies to reduce bullying prevalence in schools.

Based on our research findings, some questions have been answered, but some new questions have emerged that may be points of interest for future studies. The weaknesses of this study were represented by the sampling size and the number of experimental groups, as well as the number of educational stages and courses included. For this reason, it would be interesting to implement this programme with other types of courses and educational stages in order to know their effect. We consider that one of the main strengths of the study was the 9-month duration of the programme; in future studies it would be ideal to continue using this period of time. Furthermore, optimal outcomes would be founded not only to study the gender variable, but also the identity the student adopts regarding his gender.

The use of qualitative techniques can be particularly useful to identify areas that may be inadvertently neglected due to the researcher’s ignorance: for example, new social networks, spaces or apps where students engage in bullying behaviour. For this reason, it would be interesting to carry out an ethnographic study to observe and record the behaviours and actions of students outside of class times during school hours (entrance, recess and exit from school).

Finally, we must consider the importance of repeating this study in specialised educational centres, such as music or art centres, where artistic expression is significantly integrated into the relationships between students. In this context, the use of communicative competence as a factor in dramaturgy processes could serve as a variable that regulates the levels of bullying prevalence.

## Figures and Tables

**Table 1:** Identification of research variables.

Variables	Measurement Level	Categories
Group	Dichotomous nominal	Control Experimental
Gender	Dichotomous nominal	Male Female
Questionaries’ collection	Dichotomous nominal	Pre-test Post-test

Source: Prepared by the authors.

**Table 2:** Scale of aggression and conflict in the classroom by dimensions.

Obtained Score	Level
Between 0 and 10 points	Low aggression
Between 10 and 20 points	Medium aggression
Between 30 and 44 points	High aggression

Source: Prepared by the authors.

**Table 3:** Intimidation dimension.

Questionnaire Items	Before	After
- Would you be able to intimidate a partner? (4 points)	1.73 ± 0.96	1.69 ± 0.93
- How often have you bullied a partner? (4 points)	1.38 ± 0.70	1.34 ± 0.55
- How do you feel when a partner laughs at you? (6 points)	1.83 ± 1.47	1.58 ± 1.03
- When do you mess with a partner? What do the other colleagues do? (4 points)	1.62 ± 1.05	1.50 ± 0.91
- How often have you put or called someone for their nickname? (6 points)	3.23 ± 1.80	2.90 ± 0.98
- What do you usually do when one partner intimidates another? (5 points)	2.46 ± 1.27	2.28 ± 1.07

Source: Prepared by the authors.

**Table 4:** Communication skills dimension.

Questionnaire Items	Before	After
- Write and analyse texts about feelings, desires, perceptions regarding bullying or teasing at school; do you think this would help to diminish or solve them? (5 points)	3.71 ± 1.01	2.57 ± 0.92
- Talk and listen to your classmates about bullying or teasing at school; do you think it would help to reduce or solve them? (5 points)	3.96 ± 0.79	2.96 ± 1.32
- Do readings about bullying or teasing at school and analyse them together in class; do you think this would help to reduce or solve them? (5 points)	4.00 ± 1.15	2.46 ± 0.96

Source: Prepared by the authors.

**Table 5:** Comparison between pre-test and post-test in the experimental group.

Dimensions		M	SD	CA	KC	Shapiro-Wilk			Wilcoxon	
						SW	gl	sig	Z	*p*
Dimension 1 (29 points)	Pre-test	11.90	4.67	1.38	1.12	0.824	29	0.000	−2.712	0.007
	Post-test	10.79	4.05	1.26	1.49	0.873	29	0.002		
Dimension 2 (15 points)	Pre-test	11.72	1.98	0.29	−1.05	0.920	29	0.030	−4.365	0.001
	Post-test	8.14	2.39	0.21	0.67	0.961	29	0.356		
Total (44 points)	Pre-test	23.62	5.56	1.32	1.90	0.887	29	0.005	−4.565	0.001
	Post-test	18.93	4.96	0.98	0.75	0.910	29	0.017		

Source: Prepared by the authors. M—mean; SD—standard deviation; CA—kurtosis; KC—skewness; KS—Shapiro-Wilk; gl—degrees of freedom; sig—significance.

**Table 6:** Comparison between the control and experimental groups after applying the program.

Dimensions		M	SD	AC	KC	Shapiro-Wilk			Man-Whitney	
						KS	gl	sig	Z	*p*
Dimension 1 (29 points)	Control	12.38	3.90	0.51	−0.27	0.964	26	0.465	174,000	0.041
	Experimental	10.79	4.05	1.26	1.49	0.873	29	0.002		
Dimension 2 (15 points)	Control	11.81	2.43	−1.78	6.04	0.840	26	0.001	89,500	0.001
	Experimental	8.14	2.39	0.21	0.69	0.961	29	0.356		
Total (44 points)	Control	24.20	4.63	0.38	−0.42	0.964	26	0.478	156,000	0.001
	Experimental	18.93	4.96	0.98	0.75	0.910	29	0.017		

Source: Prepared by the authors.

**Table 7:** Comparison in the post-test and pre-test design of the experimental group, with respect to gender.

Design	Dimension	Gender	M	SD	AC	KC	Shapiro-Wilk			Mann-Whitney
							KS	gl	sig	*p*
Pre-test	Dimension 1	Male	11.93	4.92	1.35	0.84	0.821	15	0.007	0.949
		Female	11.86	4.57	1.60	2.70	0.805	14	0.006	
	Dimension 2	Male	11.47	2.07	0.51	0.85	0.910	15	0.138	0.479
		Female	12.00	1.92	0.15	1.04	0.943	14	0.457	
Post-test	Dimension 1	Male	10.67	4.81	1.57	1.88	0.776	15	0.002	0.400
		Female	10.93	3.22	0.37	0.54	0.963	14	0.767	
	Dimension 2	Male	8.20	2.14	0.50	0.26	0.933	15	0.303	0.889
		Female	8.07	2.70	0.61	0.80	0.940	14	0.415	
